# Protocol for a systematic review of sarcopenia in older adults with type 2 diabetes mellitus and its association with increased risk of mortality

**DOI:** 10.3389/fmed.2025.1505093

**Published:** 2025-09-01

**Authors:** Phatcharaphon Whaikid, Noppawan Piaseu, Anita Souza

**Affiliations:** ^1^Philosophy Program in Nursing Science (International Program), Faculty of Medicine Ramathibodi Hospital, Faculty of Nursing, Mahidol University, Bangkok, Thailand; ^2^Ramathibodi School of Nursing Center, University of Adelaide, Adelaide, SA, Australia; ^3^Faculty of Medicine Ramathibodi Hospital, Mahidol University, Bangkok, Thailand; ^4^University of Washington School of Nursing, University of Washington, Seattle, WA, United States

**Keywords:** sarcopenia, type 2 diabetes mellitus, mortality risk, systematic review, meta-analysis, protocol

## Abstract

**Background:**

Sarcopenia and type 2 diabetes mellitus (T2DM) are prevalent health conditions that significantly impact mortality risk, particularly among older adults. While both conditions have been individually associated with increased mortality, limited evidence exists regarding their combined effect, and no prior systematic review has synthesized this association specifically among older adults with T2DM. This study aims to examine the association between sarcopenia and all-cause mortality in older adults with T2DM. It seeks to evaluate whether this relationship varies by population characteristics, sarcopenia definitions, and follow-up duration.

**Methods:**

We will conduct a comprehensive literature search using databases such as PubMed, Scopus, CINAHL, and Embase to identify studies exploring the relationship between sarcopenia and all-cause mortality in older adults with T2DM from January 1, 2014, to September 1, 2024. Two authors will independently screen all eligible clinical studies. Statistical analyses will be conducted using JBI SUMARI software.

**Results:**

Preliminary findings will indicate the overall prevalence and mortality rate among older adults with sarcopenia and T2DM. By consolidating findings from diverse studies, this meta-analysis will provide clearer insights into how sarcopenia and T2DM interact to affect mortality risk.

**Conclusion:**

Understanding the relationship between sarcopenia and T2DM is crucial leading to developing effective interventions to reduce mortality risk and improve the quality of life in older adults. Addressing this important research gap will contribute to better healthcare practices and outcomes.

## 1 Introduction

Sarcopenia is a condition characterized by the progressive loss of skeletal muscle mass and strength (1), which can lead to physical disability (2, 3), poor quality of life (4, 5), and increased mortality (6, 7). It is also associated with an increased risk of falls, which contributes substantially to healthcare costs, accounting for over $50 billion annually in medical expenses (8). In 2016, sarcopenia was officially recognized as a disease associated with aging by the World Health Organization and was assigned an ICD-10-CM code (M62.84) (9), reinforcing its clinical relevance and importance for public health (1). The prevalence varies between 10% and 27%, depending on the classification criteria used (10). Sarcopenia is particularly prevalent in older adults and can be exacerbated by chronic conditions such as type 2 diabetes mellitus (T2DM) (10). T2DM is one of the most commonly observed conditions and a significant contributor to sarcopenia among its various etiologies. T2DM is a metabolic disorder characterized by high blood sugar levels due to insulin resistance or insufficient insulin production (11, 12).

T2DM may also contribute to skeletal muscle degradation through extracellular matrix (ECM) remodeling, which disrupts insulin signaling and promotes muscle fibrosis and dysfunction, thereby accelerating sarcopenia progression (13). The combination of sarcopenia and T2DM in older adults poses significant health risks, including an increased likelihood of falls, fractures, and overall mortality (14). Currently, despite having a growing awareness of the impact of sarcopenia on health outcomes, significant gaps remain in understanding of sarcopenia affect mortality risk in older adults with T2DM. Although studies have investigated the link between sarcopenia and T2DM, comprehensive research examining their relationship in terms of increased mortality risk among older adults remains limited. To date, no systematic reviews or meta-analyses have been conducted on this topic. Understanding this relationship is crucial, as sarcopenia may not only worsen diabetes outcomes but also increase the risk of premature mortality. In fact, sarcopenia has been associated with up to a 45% higher risk of mortality in older adults compared to those without sarcopenia (15). This gap in knowledge underscores the need for an updated systematic review on the relationship between sarcopenia and mortality. This review aims to examine the association between sarcopenia and all-cause mortality in older adults with T2DM, while considering whether this relationship varies based on the population, the definition of sarcopenia, and the duration of follow-up. This will pave the way for developing effective treatment strategies to manage both conditions concurrently, ultimately improving health outcomes and survival rates.

## 2 Methods

### 2.1 Study registration

This meta-analysis was registered with PROSPERO on 14 September 2024 (Registration number: CRD42024586761) and will follow the Preferred Reporting Items for Systematic review and Meta-Analysis Protocols (PRISMA- P).

### 2.2 The inclusion criteria

#### 2.2.1 Types of studies

Cross-sectional, longitudinal, and prospective cohort studies will be considered for inclusion, while animal studies, randomized controlled trials (RCTs), quasi-experimental designs, case reports, and review articles will be excluded.

#### 2.2.2 Types of participants

Participants will include individuals aged 60 years and above, as well as those diagnosed with type 2 diabetes mellitus.

#### 2.2.3 Types of outcomes

This study will investigate the prevalence of all-cause mortality among older adults affected by both T2DM and sarcopenia. The main outcome focus will be on mortality rates within 1–2 years, with secondary outcomes examining mortality at 5 and 10 years. This comprehensive approach aims to shed light on how the interplay of these conditions impacts overall survival and health outcomes in older adults.

### 2.3 Collection and analysis of data

#### 2.3.1 Search strategy

We will conduct a comprehensive literature search using databases such as PubMed, Scopus, CINAHL, and Embase to identify studies exploring the relationship between sarcopenia and all-cause mortality in older adults with type 2 diabetes mellitus. The following keywords were used to search the databases for relevant literature: “sarcopenia” OR “muscle mass” OR “muscle strength” OR “physical performance” AND “mortality” OR “death rate” OR “die” OR “died” OR “survival” AND “T2DM” OR “diabetes mellitus” OR “type 2 diabetes mellitus.” The search included studies published between January 1, 2014, and September 1, 2024. Titles and abstracts were screened for an initial extraction of all eligible studies.

#### 2.3.2 Selection of studies

**Figure 1:**
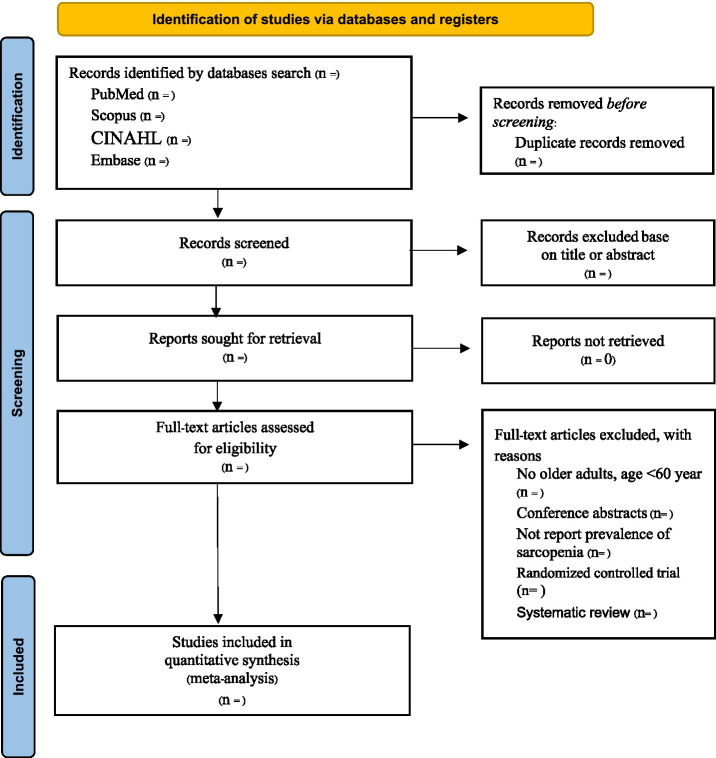
Flow diagram for Study Selection.

Two researchers will conduct a comprehensive search across all relevant databases, based on predefined inclusion and exclusion criteria to ensure the studies’ relevance and quality. Each study will be carefully cross-verified by both researchers to ensure that no pertinent studies are inadvertently excluded. This process will adhere to the PRISMA flow diagram (Figure 1), which provides a systematic framework for study selection and data extraction, ensuring a thorough and transparent review process.

#### 2.3.3 Assessment of risk of bias and quality of evidence

To ensure the credibility of the empirical evidence, the researchers will utilize the Newcastle-Ottawa Scale Quality Assessment scale (NOS) (16) to assess the risk of bias. This checklist is designed to independently evaluate the quality and rigor of studies across various research designs, including cross-sectional, longitudinal, and prospective cohort studies.

Three researchers will independently assess each study using the NOS, which involves evaluating factors such as selection of study groups, comparability of groups, and ascertainment of outcomes (or exposures). This independent assessment helps to mitigate bias and ensures that the studies included in the review meet high standards of quality and reliability. The use of the NOS checklist provides a structured and systematic approach to appraising empirical evidence, contributing to the overall validity and robustness of the research findings.

### 2.4 Statistical analysis

#### 2.4.1 Synthesis of data

The synthesis of data will involve integrating and summarizing the results from the selected studies to provide a comprehensive understanding of the factors associated with mortality. This process will include examining various aspects such as participant characteristics (e.g., sample size, sex, age, and population), definitions of sarcopenia, its prevalence, and the causes of mortality.

#### 2.4.2 Measures of effect

The authors will perform the statistical analysis using JBI SUMARI software. A meta-analysis will be conducted to investigate factors associated with the causes of mortality, utilizing a fixed-effect model through the inverse variance approach. The primary analysis will involve calculating effect sizes with 95% confidence intervals (CI) using a hazard ratio (HR) and odds ratios (OR) of mortality will be calculated.

#### 2.4.3 Assessment of heterogeneity

Heterogeneity among studies will be assessed using I^2^ statistics, with the following interpretations: (1) 0–24.9% indicating minimal heterogeneity, (2) 25.0–49.9% suggesting moderate heterogeneity, (3) 50.0–74.9% representing substantial heterogeneity, and (4) 75.0–100% indicating considerable heterogeneity. Additionally, the presence of heterogeneity will be evaluated using *χ*^2^ (chi-square) *p*-values, with *p* < 0.1 signaling significant heterogeneity.

#### 2.4.4 Assessment of reporting bias

To evaluate reporting bias, both a funnel plot and the Egger test will be employed. The funnel plot will visually represent the distribution of study results, helping us identify any potential asymmetry that may indicate bias in reporting. By using both methods, we will enhance our ability to effectively identify and analyze the potential for reporting bias in the results.

#### 2.4.5 Subgroup analysis

Subgroup analyses will be performed according to the primary analysis if sufficient data are available, focusing on populations with mortality, the definition of sarcopenia, and the duration of follow-up.

## 3 Discussion

Sarcopenia and T2DM are two health conditions that significantly impact mortality risk, particularly in older adults (17, 18). Investigating the relationship between these conditions can provide valuable insights into the associated risks and guide more effective management strategies. Older people diagnosed with T2DM experience a more pronounced and accelerated decline in both muscle mass (19, 20) and muscle strength (21, 22) compared to those without diabetes. T2DM is associated with a reduction in key components used to diagnose sarcopenia. Therefore, sarcopenia and T2DM coexist, the risk of mortality increases significantly. Individuals with both conditions tend to have poorer overall health and face a higher likelihood of severe complications and mortality (23, 24). This combination leads to a marked decline in physical health, further elevating the chances of serious complications and mortality.

Simultaneously studying sarcopenia and T2DM is essential for developing effective health management strategies that reduce mortality risk and improve the quality of life in older adults. A deeper understanding of the relationship between these conditions can drive more impactful research and enhance medical practices in caring for older adults. Current studies on sarcopenia and T2DM present mixed findings and fail to offer a clear understanding of the relationship between these conditions and mortality risk. A meta-analysis could integrate the available data, reduce heterogeneity, and enhance the precision of risk assessment. This approach would also provide greater insight into the combined effects of sarcopenia and T2DM on mortality, addressing an important research gap that requires further investigation.

However, several limitations should be considered. First, variations in the diagnostic criteria for sarcopenia (e.g., EWGSOP2, and AWGS) across studies may introduce heterogeneity that could affect the comparability of results. Second, differences in population characteristics, such as ethnicity and healthcare settings, may limit the generalizability of the findings. Third, the inclusion of only English-language publications may lead to language bias. Lastly, publication bias and the inherent limitations of observational studies (e.g., residual confounding) may also influence the pooled estimates.
